# The CEA/CD3-Bispecific Antibody MEDI-565 (MT111) Binds a Nonlinear Epitope in the Full-Length but Not a Short Splice Variant of CEA

**DOI:** 10.1371/journal.pone.0036412

**Published:** 2012-05-04

**Authors:** Li Peng, Michael D. Oberst, Jiaqi Huang, Philip Brohawn, Chris Morehouse, Kristen Lekstrom, Patrick A. Baeuerle, Herren Wu, Yihong Yao, Steven R. Coats, William Dall’Acqua, Melissa Damschroder, Scott A. Hammond

**Affiliations:** 1 Department of Antibody Discovery and Protein Engineering, MedImmune LLC, Gaithersburg, Maryland, United States of America; 2 Preclinical Oncology, MedImmune LLC, Gaithersburg, Maryland, United States of America; 3 Translational Sciences, MedImmune LLC, Gaithersburg, Maryland, United States of America; 4 Amgen Research (Munich) GmbH, Munich, Germany; University of Cincinnati College of Medicine, United States of America

## Abstract

MEDI-565 (also known as MT111) is a bispecific T-cell engager (BiTE®) antibody in development for the treatment of patients with cancers expressing carcinoembryonic antigen (CEA). MEDI-565 binds CEA on cancer cells and CD3 on T cells to induce T-cell mediated killing of cancer cells. To understand the molecular basis of human CEA recognition by MEDI-565 and how polymorphisms and spliced forms of CEA may affect MEDI-565 activity, we mapped the epitope of MEDI-565 on CEA using mutagenesis and homology modeling approaches. We found that MEDI-565 recognized a conformational epitope in the A2 domain comprised of amino acids 326–349 and 388–410, with critical residues F^326^, T^328^, N^333^, V^388^, G^389^, P^390^, E^392^, I^408^, and N^410^. Two non-synonymous single-nucleotide polymorphisms (SNPs) (rs10407503, rs7249230) were identified in the epitope region, but they are found at low homozygosity rates. Searching the National Center for Biotechnology Information GenBank® database, we further identified a single, previously uncharacterized mRNA splice variant of CEA that lacks a portion of the N-terminal domain, the A1 and B1 domains, and a large portion of the A2 domain. Real-time quantitative polymerase chain reaction analysis of multiple cancers showed widespread expression of full-length CEA in these tumors, with less frequent but concordant expression of the CEA splice variant. Because the epitope was largely absent from the CEA splice variant, MEDI-565 did not bind or mediate T-cell killing of cells solely expressing this form of CEA. In addition, the splice variant did not interfere with MEDI-565 binding or activity when co-expressed with full-length CEA. Thus MEDI-565 may broadly target CEA-positive tumors without regard for expression of the short splice variant of CEA. Together our data suggest that MEDI-565 activity will neither be impacted by SNPs nor by a splice variant of CEA.

## Introduction

Carcinoembryonic antigen (CEA; CEACAM5; CD66e) is a glycosylated human oncofetal antigen that belongs to the CEA-related cell adhesion molecule (CEACAM) family of the immunoglobulin (Ig) gene superfamily [1], [2]. CEA is closely related to CEACAM1, CEACAM3, CEACAM4, CEACAM6, CEACAM7, and CEACAM8. Carcinoembryonic antigen has been suggested to mediate cell-cell adhesion, facilitate bacterial colonization of the intestine, and protect the colon from microbial infection by binding and trapping infectious microorganisms [3].

CEA is expressed at low levels in normal tissues of epithelial origin in a polarized manner; found only at the luminal portion of the cell but not at the basolateral surface [3]. In contrast, expression of CEA is frequently high in carcinomas, including colon, pancreatic, gastric, esophageal, lung, breast, uterine, ovarian, and endometrial cancers [3]. Cancer cells not only lose polarized (luminal) expression of CEA, but actively cleave CEA from their surface by phospholipases, an action that results in serum concentrations of CEA that can approach 5 µg/mL [3], [4], [5]. Serum CEA levels may be monitored to detect a response to anti-cancer therapy, or disease recurrence in colorectal cancer [6], and serves as a prognostic indicator in patients with gastrointestinal cancers, where elevated levels indicate a poor prognosis and correlate with reduced overall survival [7], [8], [9].

Cell-bound CEA has served as a target for tumor imaging and anti-cancer therapies. Clinical studies have demonstrated that radiolabeled anti-CEA antibodies and antibody fragments, such as the Food and Drug Administration-approved, Tc-99m-labeled, anti-CEA Fab arcitumomab (CEA-Scan®), can be successfully used as imaging reagents to specifically localize CEA-expressing solid cancers [10], [11], [12]. Anti-CEA radio-immunoconjugate antibodies have also been shown to be potentially efficacious for the treatment of patients with metastatic colorectal cancer [13]. In addition, CEA-specific antibody-directed enzyme prodrug therapy and CEA-based vaccine approaches have been developed to treat cancers [14], [15].

As a novel CEA-directed therapy, we have developed a CEA-targeting bispecific single-chain antibody of the bispecific T-cell engager (BiTE) class termed MEDI-565 (also known as MT111) [16], [17], [18]. BiTE antibodies are a unique subclass of bispecific antibodies that contain one single chain variable fragment (scFv) with specificity for a tumor associated antigen molecularly fused to another scFv with specificity for CD3 on T cells [19]. Highly potent and specific tumor cell lysis is triggered only when BiTE antibodies bind both epitopes simultaneously, resulting in directing T cells to the tumor cells and activating the T cell through the CD3/T cell receptor (TCR) complex [20]. Notably, activation of T cells is independent of TCR specificity, peptide antigen presentation, and co-stimulatory signals [21]. As a result of T cell activation, granzymes and perforin are mobilized to the tumor cell-T cell interface and mediate an apoptotic killing of target cells; FAS ligand expression may also contribute to the induction of apoptosis through engagement of FAS on tumor cells [22], [23], [24]. BiTE antibodies activate both CD4+ and CD8+ T cell subsets [23], [25], [26], [27], [28]; both subsets of T cells contribute to tumor cell killing at relatively low effector T cell:target tumor cell (E:T) ratios [22], [29]. Cytokine secretion and the upregulation of cell surface IL-2 receptor by T cells (measured experimentally using antibodies recognizing the IL-2Rα chain/CD25) occur simultaneously and accompany tumor cell killing [18], [22], [25]. Both potentiate further T cell activation and proliferation, facilitating the subsequent engagement and killing of additional tumor cells bound by a BiTE antibody. BiTE antibodies not only induce the potent lysis of human cancer cell lines in vitro, but have also been shown to mediate the killing of primary human tumor cells by cancer patient T cells ex vivo [17], [25], [30]. Furthermore, BiTE antibodies promote in vivo tumor regression in numerous pre-clinical animal models [16], [31], [32], [33], [34], [35], [36] and have shown signs of clinical benefit in patients with cancer [37], [38], [39].

MEDI-565 itself is composed of a humanized single-chain antibody recognizing human CEA connected by a short flexible linker to a de-immunized single-chain antibody specific for CD3, and shares the characteristics of the BiTE antibody class as described above [16], [17], [18]. It specifically binds to CEA and not to other CEACAM family members [16]. Upon concurrent binding to CEA on cancer cells and CD3 on T cells, MEDI-565 mediates T cell activation and the subsequent killing of CEA-expressing target cells in a perforin- and granzyme-dependent manner that is insensitive to concentrations up to 5 µg/mL of soluble CEA [16], [17], [18]. Furthermore, intravenous and subcutaneous administration of MEDI-565 had anticancer activity in severe combined immunodeficient mouse xenograft models of various human cancers [18]; growth inhibition of the cancers was contingent on both the presence of un-stimulated human T cells and the expression of CEA on the cancer cells [18]. MEDI-565 is currently in phase I clinical trials (clinicaltrials.gov identifier: NCT01284231) for the treatment of gastrointestinal adenocarcinomas.

To understand the molecular basis of human CEA recognition by MEDI-565 and the impact of amino acid polymorphisms and splice variants of CEA on the activity of MEDI-565, we sought to identify the CEA domain and the critical residues involved in MEDI-565 binding. The CEA-specific arm of MEDI-565 is a humanized version of the murine antibody A5B7 [40], [41] the binding epitope of which is unknown [40], [42]. We mapped its epitope using mutagenesis and computational modeling approaches. We further investigated what impact a CEA splice variant which lacks a large portion of the identified epitope could have on the binding and activity of MEDI-565.

## Materials and Methods

### Vectors Expressing Full-length and Splice Variant CEA

The plasmid containing the full-length CEA sequence was purchased from Open Biosystems (Thermo Fisher Scientific, Huntsville, AL). The CEA complementary DNA (cDNA) was sub-cloned using sequence specific primers into a modified version of the lentiviral vector pCDH1-CMV-MCS-EF1-Puro (System Biosciences, Mountain View, CA) in which the puromycin-resistance casette was replaced with a blasticidin-resistance cassette (pCDH1-CMV-MCS-EF1-Blast) and the CEA sequence was verified by DNA sequencing. The plasmid containing the sequences of the CEA splice variant (cDNA clone DKFZp781M2392) was purchased from ImaGenes GmbH (Source BioScience UK Limited, Nottingham, UK) in association with B Bridge International (Cupertino, CA). The CEA splice variant was cloned into the puromycin resistance lentiviral vector pCDH1-HCS1-EF1-Puro (System Biosciences) using sequence-specific primers and the resulting clones were sequence-verified.

### Cell Culture, Antibodies, and Reagents

FreeStyle human embryonic kidney (HEK293) F cells (American Type Culture Collection, Manassas, VA) were cultured in FreeStyle 293 expression medium (Life Technologies/Invitrogen, Carlsbad, CA). Dihydrofolate reductase deficient (DHFR-) Chinese hamster ovary (CHO) cells or CHO DHFR- cells (American Type Culture Collection, Manassas, VA) were cultured in RPMI 1640 media (Invitrogen) containing 10% fetal bovine serum (FBS; Invitrogen) in a humidified cell culture incubator at 37°C and 5% CO_2_. CHO cells expressing the CEA splice variant (CHO SV CEA) were created by infection of CHO DHFR- cells with the pCDH1-HCS1-EF1-Puro-CEA splice variant lentiviral vector, and selected by culturing in medium containing 5 µg/mL puromycin (EMD Chemicals Group, Merck KGaA, Darmstadt, Germany) for 72 hours. CHO cells expressing the full-length CEA sequence (CHO FL CEA) were created by infecting CHO DHFR- cells with the lentiviral expression vector pCDH1-CMV-MCS-EF1-Blast-CEA, and selected by culturing in medium containing 10 µg/mL blasticidin (Invitrogen) for 10 days. Likewise, cells expressing both the CEA splice variant and full-length CEA (CHO FL+SV CEA) were created by infecting CEA splice variant-expressing cells with the pCDH1-CMV-MCS-EF1-Blast-CEA lentiviral vector followed by blasticidin selection. The anti-CEACAM5 specific monoclonal antibody (mAb) clone 26/3/13 was purchased from Genovac GmbH (Aldevron, Freiburg, Germany). Propidium iodide (PI) was purchased from Sigma-Aldrich (St. Louis, MO). Construction and production of MEDI-565 and the Control BiTE (MEC14 BiTE) has been described [17].

### Primary Pancreatic Tumor Tissues

Twenty pancreatic primary tumors were purchased from Asterand, Inc (Detroit, MI). The panel included 13 adenocarcinomas (stage I-IV), 5 pancreatic endocrine tumors and 2 benign pancreatic adenomas. Four of the 20 pancreatic tumor tissue samples had matched normal adjacent pancreatic tissue samples. Patient age ranged from 23 to 77 years. All samples were freshly frozen and collected before the initiation of any cancer treatment. Tumor samples were macrodissected to remove normal tissue, and tumor purity in all samples was greater than 85%. Normal samples were macrodissected to remove non-glandular tissue.

### Cancer Tissue cDNA Arrays

Cancer tissue cDNA arrays: HCRT101 (Colon Cancer), HGRT101 (Gastroesophageal Cancer), PNRT101 (Pancreatic Cancer), HLRT101 (lung cancer), and BCRT101 (breast cancer) were purchased from OriGene Technologies (Rockville, MD). Each array contains cDNAs from 5 to 8 normal tissues and 19 to 42 cancer tissues. The tumor stage ranged from stage I to IV and the tumor tissues were comprised of 50–90% tumor.

### Western Blotting

Cells were lysed in RIPA lysis buffer (Boston Bioproducts, Ashland, MA) and the resulting protein was quantified using the BCA protein assay kit (Thermo Fisher Scientific, Huntsville, AL). Cell lysates were mixed with NuPAGE® LDS 4X LDS sample buffer (Invitrogen) to 1X concentration; ten micrograms of total protein from each lysate was analyzed on a pre-cast NuPAGE® Novex® 10% polyacrylamide Bis-Tris gel (Invitrogen) together with the Novex® Sharp Pre-stained Protein Standard (Invitrogen) using NuPAGE® Tris-acetate SDS running buffer (Invitrogen). Protein from each gel was transferred to a polyvinylidene fluoride membrane using an iBlot® Dry Blotting System (Invitrogen) and 2X NuPAGE® transfer buffer (Invitrogen) containing 20% methanol and blocked with 3% bovine serum albumin (Sigma) in PBS pH 7.4 (Invitrogen). To detect CEA, each blot was probed overnight with 1 µg/mL of the CEACAM5-specific mAb clone 26/3/13 (Genovac), washed with PBS pH 7.4 containing 0.1% Tween-20, and subsequently probed for 1 hour with a horseradish peroxidase-conjugated donkey anti-mouse IgG secondary antibody (Jackson ImmunoResearch Laboratories, West Grove, PA). After 5 washes with PBS pH 7.4 containing 0.1% Tween-20, each blot was exposed to SuperSignal West Pico Chemiluminescent Substrate (Thermo Fisher Scientific) and exposed to BioMax MS Kodak film (Sigma) for detection of chemiluminescence resulting from bound anti-CEACAM5 antibody. Equal amounts of protein loaded into each lane of the gel were controlled by detecting the glyceraldehyde 3-phosphate dehydrogenase (GAPDH) protein using a rabbit anti-human GAPDH polyclonal antibody (Trevigen, Gaithersburg, MD) followed by a horseradish peroxidase-conjugated donkey anti-rabbit IgG (Jackson ImmunoResearch).

### Generation and Characterization of Deletion and Swap Mutants of CEA

**Figure 1:**
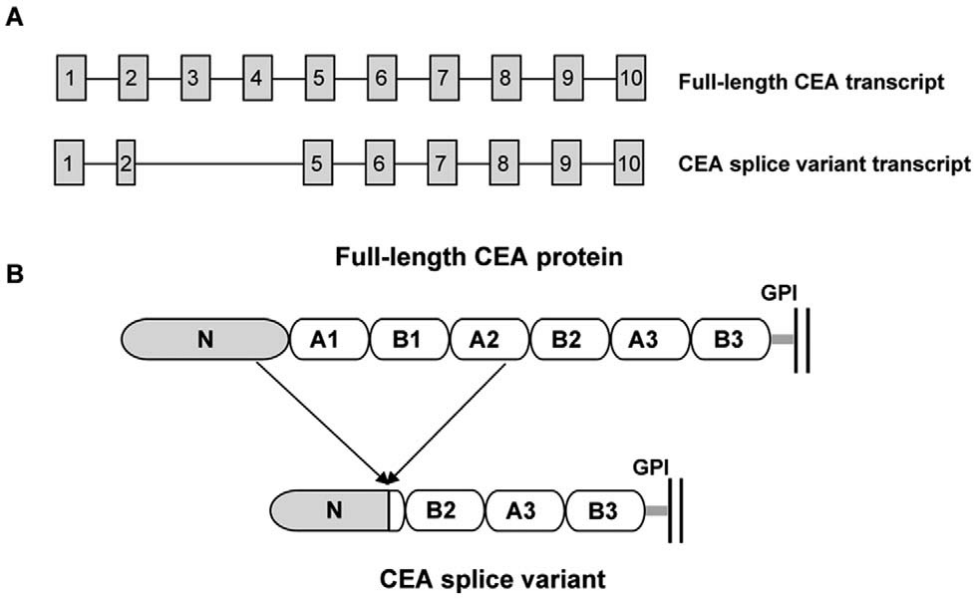
Schematic representations of the full-length CEA and CEA splice variant. A, intron and exon structure of the full-length CEA and CEA splice variant transcripts. The full-length CEA transcript (NCBI accession number NM002483) consists of 10 exons and 9 introns as shown schematically. The transcript corresponding to the CEA splice variant (NCBI accession number CR749337) removes exons 3 and 4 and a portion of exon 2 to create an alternative splice variant CEA transcript with 8 exons and 7 introns. Boxes indicate exons and lines indicate introns (not drawn to scale). B, protein domain structure of the translated full-length and splice variant transcripts of CEA. The mature full-length CEA is composed of one N-terminal domain and six IgC-like domains denoted A1, B1, A2, B2, A3, and B3. The gray bar represents the GPI linkage of the proteins to the plasma membrane shown as two parallel vertical black lines.

**Figure 2:**
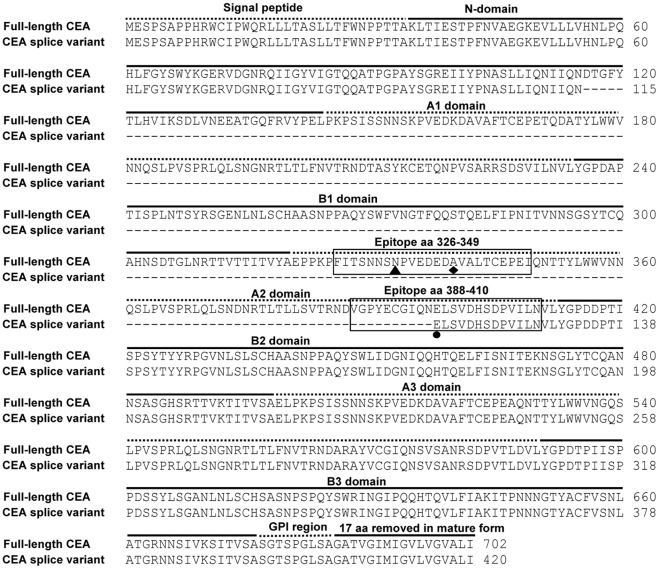
Amino acid sequence alignment of the full-length CEA (Swiss-Prot P06731-1) and CEA splice variant proteins. Amino acids corresponding to the signal peptide, the six IgC-like domains, and the C-terminal peptide which is removed during the protein maturation process are over-lined with alternating solid or dashed lines. The epitope regions (aa 326–349 and 388–410) are enclosed by black boxes. The critical aa N^333^ involved in MEDI-565 binding is labeled with an arrow▴. Two polymorphisms in the epitope region are marked with a diamond ♦ (rs10407503; aa change Ala → Asp) and a circle • (rs7249230; aa change Glu → Lys).

**Figure 3:**
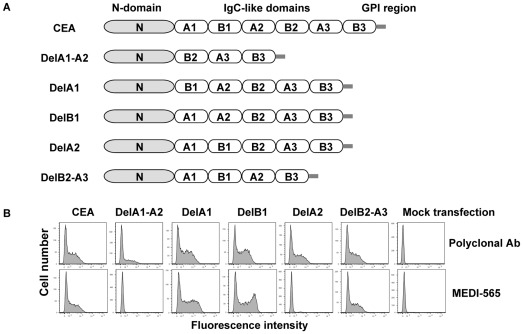
Deletion mutants of human CEA. A, linear display of the modular structures of human CEA and deletion mutants. Five deletion mutants were constructed by removing individual IgC-like domains as denoted in their name. B, flow cytometry analysis of binding of MEDI-565 to deletion mutants expressed on the surface of HEK293 F cells. All mutants were expressed well as monitored by an anti-CEA polyclonal antibody. MEDI-565 did not recognize any of the deletion mutants which lack the A2 domain (DelA1-A2 and DelA2), but bound well to all mutants which are comprised of the A2 domain (DelA1, DelB1 and DelB2-A3).

**Figure 4:**
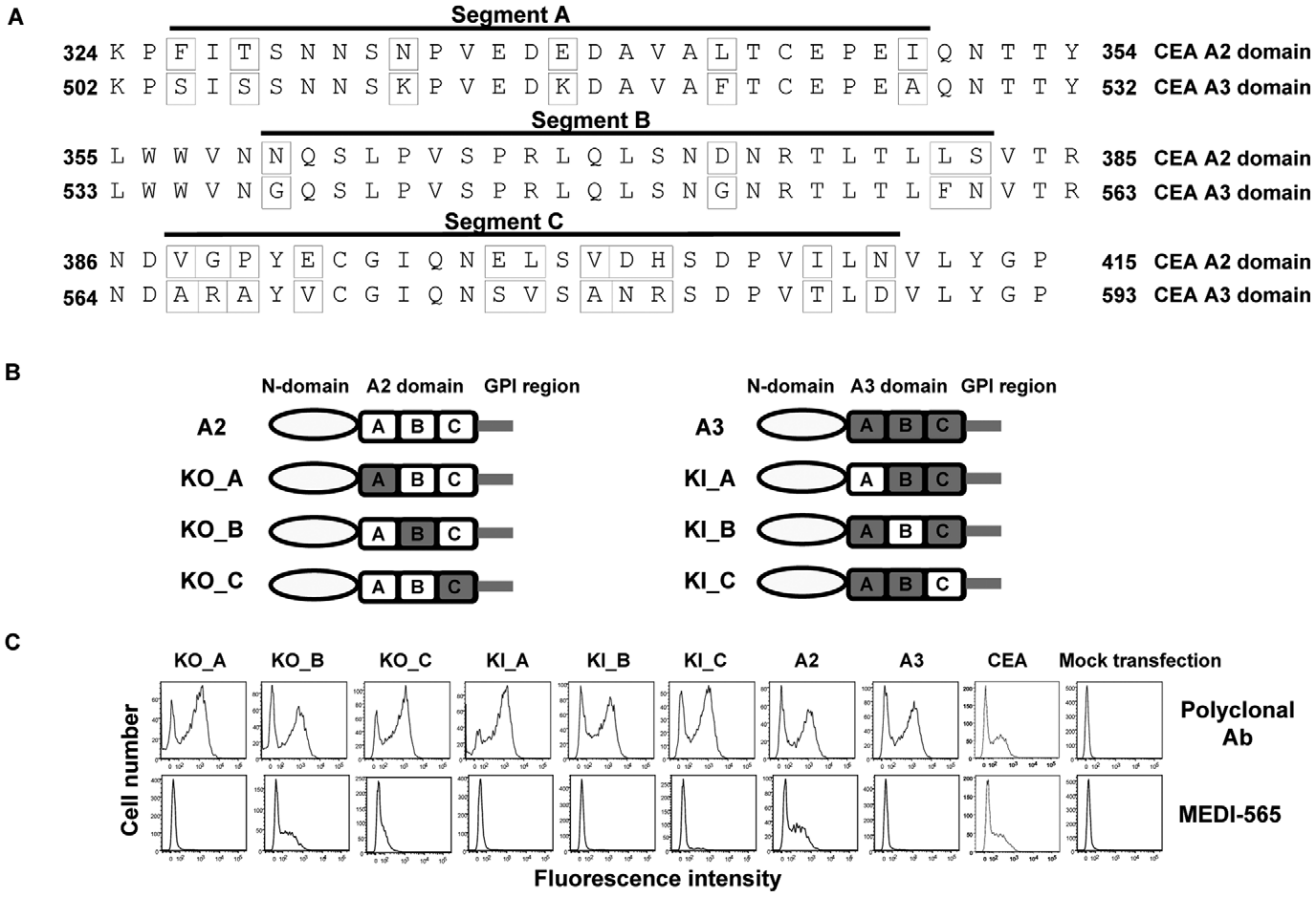
Swap-mutants of human CEA. A, amino acid sequence alignment of the A2 and A3 domains of CEA. Sequence homology analysis revealed 21 amino acids that differed between these two domains (amino acids boxed). Three segments, A, B, and C, were defined in the A2 and A3 domains to generate swap-mutants. B, a schematic display of swap mutants that were constructed by exchanging segments A, B, or C between the A2 (open boxes) and A3 (grey boxes) domains using the truncated mutant A2 or A3 as a template which encodes the N-domain, the A2 or A3 domain, and the GPI region. C, flow cytometry analysis of binding of MEDI-565 to deletion mutants expressed on the surface of HEK293 F cells. All mutants were expressed well as monitored by anti-CEA polyclonal antibody. MEDI-565 did not bind well to any of the knock-out (KO) or knock-in (KI) mutants which lack either the A or C segment of the A2 domain (KO_A, KO_C, KI_A, KI_B and KI_C), but bound well to the one variant which encoded both the A and C segments of the A2 domain (KO_B).

The cDNA encoding the CEA deletion variants DelA1-A2, Del A1, DelB1, DelA2, and DelB2-A3 and the swap variants KO_A, KO_B, KO_C, KI_A, KI_B, and KI_C (see Figures 1, 2, 3, and 4 for domain definition and variant nomenclature) were assembled and amplified by overlapping extension polymerase chain reaction (PCR) using an in-house full-length CEA cDNA clone, pNEO human CEA-GPI, as a template. The CEA variants and the full-length CEA cDNA were cloned into a mammalian expression vector pcDNA3.1 (Invitrogen).

Expression vectors encoding the CEA deletion and swap mutants were transiently transfected for expression of glycophosphatidyl inositol (GPI)-anchored proteins using FreeStyle HEK293 F cells (Invitrogen). One day prior to transfection, HEK293 F cells were seeded at a density of 0.7×10^6^ cells/mL. Three and a half micrograms of each expression vector were transfected into 5 mL of a suspension of HEK293 F cells using 5 µL of 293fectin transfection reagent (Invitrogen). Transfected cells were incubated with 5 µg/mL of MEDI-565 for 1 hour. For the detection of bound MEDI-565, which possesses a six-histidine tag at its carboxy-terminus, the cells were washed three times with PBS and incubated with 1 µg/mL of an Alexa Fluor® 488-conjugated anti-penta-His antibody (Qiagen, Valencia, CA) for 30 minutes, and then analyzed for binding using the LSRII flow cytometer (BD Biosciences, San Jose, CA). Expression of CEA deletion mutants was monitored with a goat anti-CEA polyclonal antibody (R&D Systems, Minneapolis, MN) that was detected by an R-phycoerythrin-conjugated anti-goat IgG (Invitrogen).

### Site-directed Mutagenesis and Computational Homology Modeling

Site-directed mutagenesis and computational homology modeling were implemented to identify amino acids within CEA that are critical for MEDI-565 binding. The following amino acids in the epitope-containing segments A and C of the A2 domain (see Results and Discussion section) that differ from those in the A3 domain were mutated in clusters or individually to encode the corresponding amino acids of the A3 domain: F^326^T^328^N^333^ (KO_FTN), N^333^E^338^ (KO_NE), N^333^ (KO_N), E^338^L^343^I^349^ (KO_ELI), V^388^G^389^P^390^E^392^ (KO_VGPE), E^398^L^399^V^401^D^402^H^403^ (KO_ELVDH), and I^408^N^410^ (KO_IN). A modeled structure of the A2 domain of CEA was constructed using SWISS-MODEL workspace [43] and the crystal structure of mouse CEACAM1A (33.7% sequence identity with mature human CEA protein, NCBI accession number NP_001020083.1, Protein Data Bank accession number 1L6Z) [44]. This model was used as a template to engineer two additional mutants. These two mutants 1) substituted clusters V^388^G^389^P^390^E^392^ and I^408^N^410^ of the A2 domain with the corresponding clusters of the A3 domain (KO_VGPE+IN), and 2) grafted clusters F^326^T^328^N^333^, V^388^G^389^P^390^E^392^, and I^408^N^410^ of the A2 domain into the A3 domain (KI_FTN+VGPE+IN), respectively. All mutants were assembled by overlapping extension PCR using the truncated mutants A2 or A3 as a template, which encodes for the N-terminal domain, the A2 or A3 domain, and the GPI region. They were cloned into the mammalian expression vector pcDNA3.1 (Invitrogen). Expression of these mutants and binding analyses were conducted as listed above for the deletion and swap mutants.

### Design of Real-time Quantitative PCR Primer and Probes for Full-length and Splicing Variant of CEA

Full-length mRNA transcript sequences for CEA (NM_004363.2) and the CEA splice variant (DKFZp781M2392) were retrieved from the NCBI Reference Sequences database. For the full-length CEA assay we targeted the splice junctions of exons 3 and 4 to design the gene specific probe. For the CEA splice variant assay we targeted the splice junctions of exons 2 and 5 exon to design the gene specific probe. All primer/probes were imported into the Primer Express (Applied Biosystems, Foster City, CA) software tool to ensure the optimal design for utilization in the TaqMan Gene Expression assay procedure. All probes were designed to incorporate a minor groove binding moiety (MGB), and were labeled with a fluorescent dye (FAM) for detection and as a non-fluorescent quencher. Primers and probes were custom ordered from Applied Biosystems. Sequences for all primer/probe combinations are as follows:

CEA full-length: The sequence of the probe is 5′-CAGGCGCAGTGATTCA-3′.

The sequence of the forward primer 5′-GAAACCCAGAACCCAGTGAGT- 3′.

The sequence of the reverse primer is 5′ GCCATAGAGGACATTCAGGATGAC-3′.

CEA splicing variant: The sequence of the probe is; CEA 5′-ATGCATCCCTGCTGATCC-3′.

The sequence of the forward primer is 5′-CGCATACAGTGGTCGAGAGATAATA-3′.

The sequence of the reverse primer is 5′-CGCTGTGGTCAACACTTAATTTGT-3′.

### Real-Time Quantitative PCR from Frozen Pancreatic Tissue Specimens

Real-time qPCR was conducted with the BioMark™ Dynamic Array Microfluidics System (Fluidigm Corporation, South San Francisco, CA) using RNA isolated from frozen pancreatic tissues. First, total RNA was extracted from frozen slide sections of pancreatic tissue classified as normal (NAT, normal adjacent to tumor), adenocarcinoma, benign adenoma, or endocrine tumor using the ZR RNA MicroPrep kit (Zymo Research, Orange, CA). RNA purity and concentration were determined spectrophotometrically. RNA quality was assessed on an Agilent 2100 Bioanalyzer using the RNA 6000 Nano LabChip® (Agilent Technologies, Santa Clara, CA). Single stranded cDNA was generated from total pancreas RNA using the SuperScript® III First-Strand Synthesis SuperMix (Invitrogen). cDNA samples were pre-amplified using TaqMan Pre-Amp Master Mix (Invitrogen), according to the manufacturer’s instructions. Reactions were cycled with the recommended program for 14 cycles and then diluted 1∶5 with TE buffer. Pre-amplified cDNA was either utilized immediately or stored at −20°C until processed. Samples and TaqMan Gene Expression assays (Applied Biosystems, Carlsbad, CA) were load onto a 96.96 dynamic array (Fluidigm) according to the manufacturer’s instructions. The prepared array was loaded on the BioMark™ Real-Time PCR System (Fluidigm) for thermal cycling (10 min at 95°C followed by 40 cycles of 95°C for 15 sec and 1 min at 60°C). Upon completion of the qPCR, Fluidigm Real-Time PCR Analysis software was used to generate threshold cycle (Ct) values. A Ct cutoff value of 24, below which the samples were considered to be positive and above which the samples were considered to be negative for transcript expression, was empirically selected.

### Real-Time Quantitative PCR from Tissue cDNA Arrays

TissueScan™ Disease Tissue quantitative polymerase chain reaction (qPCR) arrays (Origene Technologies, Rockville, MD) were employed to determine the expression of full-length and CEA splice variant (SV) transcripts in normal and cancerous tissues of various stages and grades. For each tissue cDNA array, lyophilized cDNA was resuspended in 2.5 µL of ribonuclease-free water. Plates were sealed, vortexed, and centrifuged (1200 g for 1 min) to ensure resuspension of the full cDNA sample. A 0.2X mixture of the TaqMan assays (Applied Biosystems) was then created according to protocols supplied by the manufacturer for pre-amplification (one cycle of 95°C, 10 min followed by 14 cycles of 95°C 15 sec/60°C 4 min). Subsequent amplification and transcript detection (one cycle of 95°C, 20 sec followed by 40 cycles of 95°C, 1 sec/60°C, 20 sec) of the specific target transcripts was carried out using an ABI 7900HT Fast Real Time PCR Instrument (Applied Biosystems). Upon completion of the qPCR, SDS 2.2 Software (Ericsson, Stockholm, Sweden) was used to generate threshold cycle (Ct) values. A Ct cutoff value of 30, below which the samples were considered to be positive and above which the samples were considered to be negative for transcript expression, was empirically selected. As positive controls, cDNAs from CHO cells expressing either full-length CEA (CHO FL CEA) or the CEA splice variant (CHO SV CEA) were used. Complementary DNA isolated from DHFR-deficient CHO cells lacking CEA expression was used as a negative control for qPCR.

### Antibody Binding Assays

For binding studies with MEDI-565, Control BiTE (bispecific single-chain antibody specific for the herbicide mecoprop and human CD3; also known as MEC14 BiTE), anti-CEACAM5 mAb clone 26/13/3 (Genovac), or control mouse IgG1 (BioLegend, San Diego, CA) were used. The antibodies were incubated with CHO cells at a concentration of 10 µg/mL diluted in PBS/2% FBS for 20 minutes at 4°C. For the detection of bound MEDI-565 and Control BiTE, the CHO cells were washed three times with PBS/2% FBS and incubated with 4 µg/mL of an AlexaFluor® 488-conjugated anti-penta-His antibody (Qiagen) in PBS/2% FBS for 15 minutes at 4°C in the dark. For the detection of bound anti-CEACAM5 and control IgG mAbs, CHO cells were washed three times with PBS/2% FBS and incubated with 10 µg/mL of an Alexa Fluor® 488-conjugated goat anti-mouse IgG (Invitrogen) in PBS/2% FBS for 15 minutes at 4°C in the dark. All CHO cells were washed one time with PBS/2% FBS, resuspended in PBS/2% FBS containing 10 µg/mL of PI and the fluorescence intensities of bound secondary antibodies were detected by flow cytometry using an LSRII flow cytometer (BD Biosciences); the data were analyzed using FlowJo Software (Treestar, Ashland, OR). Viable CHO cells (PI negative) were examined for the fluorescence intensities of bound secondary antibodies. Non-linear regression analyses employing one-site saturation binding models using mean fluorescent intensity values of antibody binding to CEA-expressing cells were performed using GraphPad Prism software version 5.01 for Windows (GraphPad Software, San Diego, CA.) to determine apparent equilibrium binding constants (apparent K_D_ values) of MEDI-565 binding to CEA.

### Cellular Cytotoxicity Assays

MEDI-565 induced T-cell killing of CHO DHFR-, CHO FL CEA, CHO SV CEA and CHO FL+SV CEA cells was determined using a flow cytometry-based assay that measures the viability of dye-labeled target cells. Human CD3+ T cells were enriched from heparinized whole blood PBMCs of healthy donors by the RosetteSep® human T-cell enrichment cocktail (Stem Cell Technologies, Vancouver, BC, Canada) and RosetteSep T-cell density medium (Stem Cell Technologies) according to the manufacturer’s protocol. CHO DHFR-, CHO FL CEA, CHO SV CEA and CHO FL+SV CEA cells were used as target cells, and labeled with 3,3′-dioctadecyloxacarbocyanine perchlorate (DiO) cell labelling solution (Invitrogen) to distinguish them from effector T cells during flow cytometry analysis. Enriched CD3-positive T cells and DiO-labeled CHO cells were suspended in RPMI-1640 medium (Invitrogen) containing 10% FBS and 4.5 g/L glucose. Serial dilutions of MEDI-565 or Control BiTE (containing an scFv recognizing the herbicide mecoprop in place of the CEA-binding scFv) were added to target and effector cells in 96-well non-tissue culture treated plates (Corning, Corning, NY) at an effector-to-target ratio of 10∶1. The co-cultures were incubated at 37°C in a humidified tissue culture chamber with an atmosphere of 5% CO_2_ for 48 hours. Cytotoxicity, defined as a loss of cell membrane integrity, was monitored by cellular uptake of Propidium Iodide (PI); live cells exclude PI while dead cells do not. An LSRII flow cytometer using FACSDiva software (BD Biosciences) was used to collect data and FlowJo software (Treestar) was used to analyze the data. Cytotoxicity (specific lysis) was calculated as the percentage of all DiO-labeled target cells identified as nonviable in each well, minus background values calculated from wells containing target cells and T cells without MEDI-565. Non-linear regression analyses using a four-parameter fit were performed with GraphPad Prism software version 5.01 for Windows (GraphPad Software) to generate sigmoidal dose-response curves and determine calculated half-maximal effective concentrations (EC_50_) values. Sigmoidal dose response curves typically had R^2^ values >0.90, and EC_50_ values were used for comparison of bioactivity. CD8+ and CD4+ T cells were subdivided using a PECy7-conjugated mouse anti-human CD8 mAb and a V450-conjugated mouse anti-human CD4 mAb (BD Biosciences). T-cell activation (CD25 up-regulation) was monitored by the allophycoerytherin (APC)-conjugated mouse anti-human CD25 mAb (Becton Dickinson Biosciences).

To measure the killing of human tumor cell lines by MEDI-565 or the control BiTE, cell lines were co-cultured with enriched CD3+ T cells and BiTE antibody as described above, and cell lysis was measured by the release of caspase-3 into the tissue culture media via a caspase-3 specific electrochemiluminescence assay (Meso Scale Discovery, Gaithersburg, MD).

### Statistical Analyses

The statistical significance of differences in affinity (apparent K_D_) and cytotoxicity (EC_50_) values was calculated using two-tailed, parametric t tests calculated in GraphPad Prism software version 5.01 for Windows (GraphPad Software).

## Results

### MEDI-565 Binds a Nonlinear Epitope in the A2 Domain of CEA

MEDI-565 recognizes full length CEA; however, the corresponding epitope is unknown. CEA deletion mutants were generated to identify the domain of CEA to which MEDI-565 binds. The full-length CEA transcript (NCBI accession number NM_002483) contains 10 exons and 9 introns encoding a 702 amino acid (aa) protein composed of a 34 aa processed leader sequence, one IgV-like N-terminal domain, six immunoglobulin constant (IgC)-like domains denoted A1, B1, A2, B2, A3 and B3, and a C-terminal 17 aa peptide which is removed during GPI linkage [1], [2], [45], [46] (Figure 1, 2). Five deletion mutants were constructed by removing the following IgC-like domains: A1, B1 and A2 domains (DelA1-A2), A1 domain (DelA1), B1 domain (DelB1), A2 domain (DelA2), and B2 and A3 domains (DelB2-A3) as shown in Figure 3A. The mutants were transiently expressed as GPI-anchored proteins on HEK293 F cells. The binding of MEDI-565 or the anti-CEA polyclonal control antibody to each of the deletion mutants was analyzed using flow cytometry. MEDI-565 did not recognize any of the deletion mutants which lack the A2 domain (DelA1-A2 and DelA2, Figure 3B), but bound well to all mutants that contained it (DelA1, DelB1 and DelB2-A3, Figure 3B). Additionally, when all but the A2 IgC-like domains were removed (A2; Figure 4B), MEDI-565 still recognized the corresponding truncated CEA protein at a level similar to that measured for full-length CEA protein (Figure 4C). These results suggested that the epitope of MEDI-565 is localized in the A2 domain of CEA.

The A3 domain is not involved in MEDI-565 binding despite being highly homologous with the epitope-containing A2 domain; therefore, swap-mutants were constructed by exchanging short segments of the A2 domain with the corresponding portions of the A3 domain to further refine the MEDI-565 binding epitope. Two truncated CEA mutants (A2 and A3) were engineered and used as templates for the construction of swap mutants. The A2 or A3 mutant is comprised of the N-domain, the A2 or A3 domain, and the GPI region. An alignment of the amino acid sequences of the A2 and A3 domains identified differences in their aa composition. These regions of sequence diversity were divided into three shorter segments labeled as A (aa 326 to 349), B (aa 360 to 382) and C (aa 388 to 410) (Figure 4A). Six swap mutants were generated by swapping segments A, B or C of the A3 domain into the A2 domain (KO mutants), or by swapping segments of the A2 domain into the A3 domain (KI mutants) as shown in Figure 4B. MEDI-565 did not bind to any of the KO or KI mutants lacking either the A or C segment of the A2 domain (KO_A, KO_C, KI_A, KI_B and KI_C, Figure 4B, C), but bound well to the swap variant which encoded both the A and C segments of the A2 domain (KO_B, Figure 4B, C). Some residual binding of MEDI-565 to the KO_C mutant could be detected. Together with MEDI-565 lack of binding to KO_A mutant, this data indicated that segment C significantly contributed to MEDI-565 binding, but to a lesser degree than segment A. Due to the high degree of identity between segment B in the A2 and the A3 domains, direct involvement of this segment in MEDI-565 binding could not be entirely ruled out using a swap mutant-based approach. It is however unlikely for the following reasons 1) MEDI-565 did not bind to the mutants encoding the B segment of A2 domain in conjunction with either A or C segment of the A2 domain (KO_A or KO_C, Figure 4); 2) The binding level of MEDI-565 to the mutant encoding both the A and the C segments of A2 domain (KO_B) was comparable to the signal of both the full-length CEA and the A2 deletion mutant (Figure 4); and 3) The modeled structure of the A2 domain using murine CEACAM1 as a template revealed that segment B was spatially distal from critical residue N^333^ (see following section). Therefore, the swap-mutants revealed that MEDI-565 bound to a nonlinear epitope in the A2 domain of CEA, which is comprised of two segments, namely 326–349 (segment A) and 388–410 (segment C).

### Identification of Critical Residues of CEA that Contribute to MEDI-565 Binding

**Figure 5:**
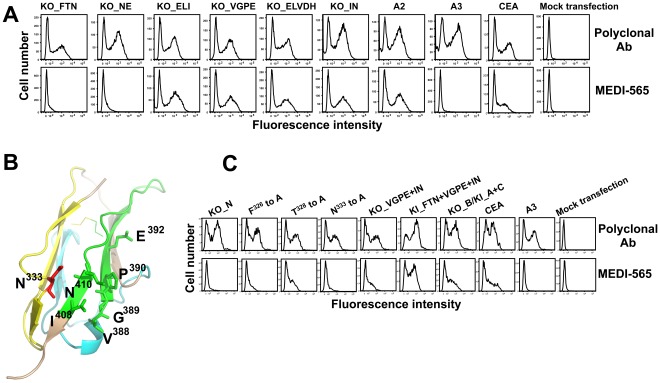
Critical amino acids in CEA involved in MEDI-565 binding. A, flow cytometry analysis of binding of MEDI-565 to CEA mutants targeting amino acids in segments A and C of the A2 domain. MEDI-565 did not bind to the variants in which the N^333^ of the A2 domain was mutated (KO_FTN and KO_NE), but bound well to the other mutants. B, a modeled structure of the A2 domain of CEA revealed two clusters of amino acids (V^388^G^389^P^390^E^392^ and I^408^N^410^) in segment C (green) that were spatially close to the critical aa N333 (red) in segment A (yellow), while segment B (cyan) was distal to aa N333. C, binding characteristics of MEDI-565 to CEA mutants with single or combinatorial mutations. Replacing N^333^ with Lys (KO_N) or Ala (N^333^ to A) disrupted the binding of MEDI-565 to the A2 domain. Mutating F^326^ or T^328^ to Ala reduced the binding of MEDI-565 (F^326^ to A, T^328^ to A). Knocking-out the amino acids V^388^G^389^P^390^E^392^ and I^408^N^410^ together from the A2 domain also reduced MEDI-565 binding (KO_VGPE+IN). Grafting of three amino acids F^326^T^328^N^333^ of segment A of the A2 domain in combination with six amino acids V^388^G^389^P^390^E^392^ and I^408^N^410^ of segment C of the A2 domain into the A3 domain (KI_FTN+VGPE+IN) resulted in MEDI-565 binding to a level comparable to that of the mutant encoding both segments A and C of the A2 domain together (KO_B/KI_A+C, KO_B encodes the same amino acids as KI_A+C).

Site-directed mutagenesis and computational homology modeling were employed to identify critical residues within segments A and C that are essential for the binding of MEDI-565. The amino acids of segments A and C of the A2 domain that differed from the A3 domains were replaced with the corresponding A3 residues encoding several substitutions at a time: F^326^T^328^N^333^ (KO_FTN), N^333^ E^338^ (KO_NE), and E^338^L^343^I^349^ (KO_ELI) V^388^G^389^P^390^E^392^ (KO_VGPE); E^398^L^399^V^401^D^402^H^403^ (KO_ELVDH) and I^408^N^410^ (KO_IN) (Figure 4A). The binding of MEDI-565 was substantially decreased to the variants in which residue N^333^ was mutated (KO_FTN and KO_NE), but bound well to the other mutants (Figure 5A). Furthermore, replacing only residue N^333^ with either its counterpart residue Lys in the A3 domain (KO_N) or with Ala (N^333^ to A) abolished MEDI-565 binding (Figure 5C). Mutating the residue F^326^ or T^328^ to Ala substantially decreased the binding of MEDI-565 (F^326^ to A, T^328^ to A), suggesting that they were also involved in the interaction with MEDI-565 but to a lesser extent than residue N^333^ (Figure 5C). Taken together, our data demonstrated the importance of F^326^, T^328^, and N^333^. Finally, since E^338^ is encoded in the KO_ELI variant which binds well to MEDI-565, we concluded that this residue is not energetically involved in MEDI-565 epitope. We had previously noted that grafting only segment A that includes the F^326^, T^328^, and N^333^ residues of the A2 domain into the A3 domain (KI_A) did not result in MEDI-565 binding. This suggested that MEDI-565 binding epitope comprises additional critical residues and pointed towards it being non-linear and conformational.

The modeled structure of the A2 domain also revealed two clusters of amino acids V^388^G^389^P^390^E^392^ and I^408^N^410^ in segment C that were spatially close to the already identified critical residue N^333^ in segment A (Figure 5B). This observation suggested that these amino acids could contribute to the binding of MEDI-565 in concert with N^333^. Knocking-out amino acids V^388^G^389^P^390^E^392^ (KO_VGPE) or I^408^N^410^ (KO_IN) separately (Figure 5A), or knocking-in the entire segment C which includes both clusters of V^388^G^389^P^390^E^392^ and I^408^N^410^ (KI_C) (Figure 4C) had no effect in abolishing or restoring MEDI-565 binding, respectively. However, knocking-out these amino acids together reduced (but did not completely abolish) MEDI-565 binding (KO_VGPE+IN, Figure 5C). Thus, amino acids V^388^, G^389^, P^390^, E^392^, I^408^, and N^410^ in segment C of CEA could also be involved in MEDI-565 binding.

It is possible that some knock-out CEA variants exhibited an incorrect fold in or near the MEDI-565 epitope region, thereby losing their binding capacity. Therefore, we further confirmed the potential epitope regions identified with knock-out variants by using gain of function (knock-in) mutants. Indeed, “knocking in” segments A (326–349) and C (388–410) of the A2 domain into the A3 domain (KO_B/KI_A+C, KO_B encodes the same amino acids as KI_A+C) resulted in MEDI-565 binding comparable to full length CEA (Figure 4C, 5C). Furthermore, only grafting the three amino acids F^326^T^328^N^333^ of segment A with the six amino acids V^388^G^389^P^390^E^392^ and I^408^N^410^ of segment C into the A3 domain (KI_FTN+VGPE+IN) also resulted in MEDI-565 binding comparable to full length CEA (Figure 5C). In summary, these results demonstrate that the epitope of CEA bound by MEDI-565 is a nonlinear, conformational epitope located in the A2 domain of CEA; it is comprised of two segments of amino acids 326 to 349 and 388 to 410 with critical amino acids F^326^, T^328^, N^333^, V^388^, G^389^, P^390^, E^392^, I^408^, and N^410^. The residue N^333^ may contribute more to the binding of MEDI-565, since mutating it alone completely disrupted the interaction (Figure 5C).

### Identification of CEA Variants

MEDI-565 binds to mature full-length CEA. However, polymorphisms and/or isoforms of CEA may alter the binding epitope and negatively affect the ability of MEDI-565 to bind. Once the epitope of CEA bound by MEDI-565 had been identified, the ability of MEDI-565 to recognize cancerous cells expressing polymorphisms and isoforms of CEA could be evaluated.

Polymorphisms of CEA were surveyed using the NCBI single-nucleotide polymorphism (SNP) database (http://www.ncbi.nlm.nih.gov/projects/SNP). Two non-synonymous coding SNPs of CEA (rs10407503, rs7249230) were identified in the binding epitope of MEDI-565 (shown in Figure 2). The single-nucleotide C to A change in the SNP rs10407503 resulted in the amino acid change of Ala to Asp at the aa position 340. The single-nucleotide A to G change in the SNP rs7249230 encoded an aa change of Glu to Lys at the aa position 398. According to the SNP database, the minor allele frequencies in the population for rs10407503 are 0.014∼0.267 and minor allele frequencies for rs7249230 are 0.03∼0.3 in the population, respectively. However, the minor allele homozygosity rate for rs10407503 is close to 0 in both European and Asian populations and is 0.083 in Sub-Saharan African populations. The minor allele homozygosity rate for rs7249230 was from 0 to 0.068 in different populations. Since the homozygosity rates of both SNPs are very low, we anticipated the identified CEA SNPs to have little or no impact on MEDI-565 binding to CEA for these populations.

In addition, the National Center for Biotechnology Information (NCBI) GenBank® database (http://www.ncbi.nlm.nih.gov/genbank) was searched for splice variants of CEA. A single splice variant (NCBI accession number CR749337) from colon cancer tissue was identified. This transcript uses an alternative splice donor site in exon 2 and skips exons 3 and 4; thus, the translation of the transcript results in a 420 aa protein with an in-frame truncation from amino acids 116 to 396 of the full-length CEA. This truncation deletes a small portion of the N-terminal domain, the entire A1 and B1 domains, and a large portion of the A2 domain (Figure 1, 2). The splice variant sequence codes for the same 34-aa processed leader sequence and C-terminal 17-aa peptide which is expected to be removed during GPI linkage in a similar manner as full-length CEA, and, after undergoing similar post-translational modifications as full-length CEA, is predicted to be a mature, GPI-anchored membrane protein of 369 amino acids.

Another putative CEA splice variant involving novel splicing of exons 9, 10 and the intervening intron sequence has been detected in the peripheral blood of colon cancer patients by reverse transcription PCR [47]. However, sequences within the middle part of a 152 bp PCR product (base pairs 26–99) of the putative CEA isoform isolated from white blood cells did not match any part of the genomic sequence of CEA. Therefore, this PCR product more likely represents a PCR artifact and not a real CEA splice form. Consequently, we did not investigate this putative CEA splice variant further.

### Expression of the CEA Splice Variant Transcript in Normal and Cancerous Human Tissues

The biological function and distribution of the CEA splice variant in normal and cancerous tissues is unknown. To determine the expression frequency of full-length and CEA splice variant transcripts in normal and cancerous human tissues, real-time qPCR was performed using both RNA isolated from frozen primary human pancreatic tissues and cDNA generated from frozen colorectal, lung, breast and pancreatic tissues purchased in the form of multi-tissue cDNA arrays. Each analysis used sequence-specific primers that specifically amplified either full-length CEA or CEA splice variant sequences.

Results (Table 1) of qPCR analysis using frozen primary pancreatic tissue specimens demonstrated that full-length CEA transcript was detected in normal human pancreatic tissues (3 of 4). Among diseased tissue, the full length transcript was found frequently in pancreatic adenocarcinoma (12 of 13) and less frequently in benign adenomas (1 of 2) and in pancreatic endocrine tumors (3 of 5), although the total number of specimens representing the latter two categories were small. Transcripts of the CEA splice variant were not detected in normal pancreatic tissues (0 of 4), benign adenomas (0 of 2) nor in endocrine tumors (0 of 5), and were rarely detected in pancreatic adenocarcinomas (1 of 13). Expression of the CEA splice variant transcript in the single positive adenocarcinoma specimen was concordant with full-length CEA transcript expression (Table S1).

**Table 1:** Percent expression of full-length CEA and CEA splice variant transcripts in primary human tissues determined by qPCR.

Tissue Type	Full Length CEA expression	CEA splice variant expression
Pancreas, Normal	3/4 (75%)	0/4 (0%)
Pancreas, Benign Adenoma	1/2 (50%)	0/2 (0%)
Pancreas, Adenocarcinoma	12/13 (92%)	1/13 (8%)
Pancreas, Endocrine Tumor	3/5 (60%)	0/5 (0%)

Results from qPCR analysis using human tissue cDNA arrays (Table 2) showed that full-length CEA transcript was commonly detected (Ct≤30) in pancreatic adenocarcinomas (4 of 5), colon (41 of 42), gastroesophageal (38 of 42), lung (39 of 40), and breast cancers (38 of 41) of various grades and stages. Full-length CEA transcripts were infrequently detected in pancreatic endocrine tumors (2 of 14). In normal colon (5 of 5), lung (8 of 8), and gastroesophageal (6 of 6) tissues, full-length CEA transcripts were expressed at relatively high levels (Ct<25). In contrast, in normal breast tissue the full-length CEA transcripts were expressed at lower levels (25≤Ct<30) in most of the samples tested (5 of 7). No expression (Ct>30) or low level (25≤Ct<30) expression of the full-length CEA transcript was found in normal pancreatic tissues (3 of 4).

**Table 2:** Percent expression of full-length CEA and CEA splice variant transcripts determined by qPCR using human tissue cDNA arrays.

Tissue Type	Full Length CEA expression		CEA splice variant expression
Pancreas, Normal	3/4 (75%)		0/4 (0%)
Pancreas, Benign Adenoma	1/2 (50%)		0/2 (0%)
Pancreas, Adenocarcinoma	12/13 (92%)		1/13 (8%)
Pancreas, Endocrine Tumor	3/5 (60%)		0/5 (0%)
Colon, normal	5/5 (100%)		5/5 (100%)
Colon, adenocarcinoma	41/42 (98%)		41/42 (98%)
Pancreas, normal	3/4 (75%)		0/4 (0%)
Pancreas, adenocarcinoma	4/5 (80%)		1/5 (20%)
Pancreas, Endocrine Tumor	2/14 (14%)		0/14 (0%)
Gastroesophageal, normal	6/6 (100%)		5/6 (83%)
Gastroesophageal, cancer	38/42 (90%)		21/42 (50%)
Lung, normal	8/8 (100%)		0/8 (0%)
Lung, cancer	39/40 (98%)		12/40 (30%)
Breast, normal	5/7 (71%)		0/7 (0%)
Breast, adenocarcinoma	38/41 (93%)		5/41 (12%)
**Tissue Type**	**Full Length CEA expression**	**CEA splice variant expression**	
Colon, normal	5/5 (100%)	5/5 (100%)	
Colon, adenocarcinoma	41/42 (98%)	41/42 (98%)	
Pancreas, normal	3/4 (75%)	0/4 (0%)	
Pancreas, adenocarcinoma	4/5 (80%)	1/5 (20%)	
Pancreas, Endocrine Tumor	2/14 (14%)	0/14 (0%)	
Gastroesophageal, normal	6/6 (100%)	5/6 (83%)	
Gastroesophageal, cancer	38/42 (90%)	21/42 (50%)	
Lung, normal	8/8 (100%)	0/8 (0%)	
Lung, cancer	39/40 (98%)	12/40 (30%)	
Breast, normal	5/7 (71%)	0/7 (0%)	
Breast, adenocarcinoma	38/41 (93%)	5/41 (12%)	

Expression (Ct≤30) of CEA splice variant transcripts within the tissue cDNA arrays was found in most colon (41 of 42) and in half of the tested gastroesophageal cancers (21 of 42). Expression was also seen in a proportion of lung (12 of 40) and breast cancers (5 of 41) of various grades and stages. Expression of the CEA splice variant transcript was infrequently found in pancreatic adenocarcinomas (1 of 5) and was not found in endocrine pancreatic cancers (0 of 14). Normal colon (5 of 5) and gastroesophageal (5 of 6) tissues showed CEA splice variant expression, but was absent in normal pancreas (0 of 4), lung (0 of 8), and breast (0 of 7). Expression of CEA splice variant transcripts, although found in fewer tumor samples than full-length CEA transcripts, was always concordant with expression of full-length CEA transcripts (Table S1). Thus, expression of the CEA splice variant transcripts varied in different cancers; however, it was always coexpressed with full-length CEA transcripts.

### The CEA Splice Variant Neither Mediates nor Interferes with MEDI-565-induced T-cell Activation and Target Cell Killing

**Figure 6:**
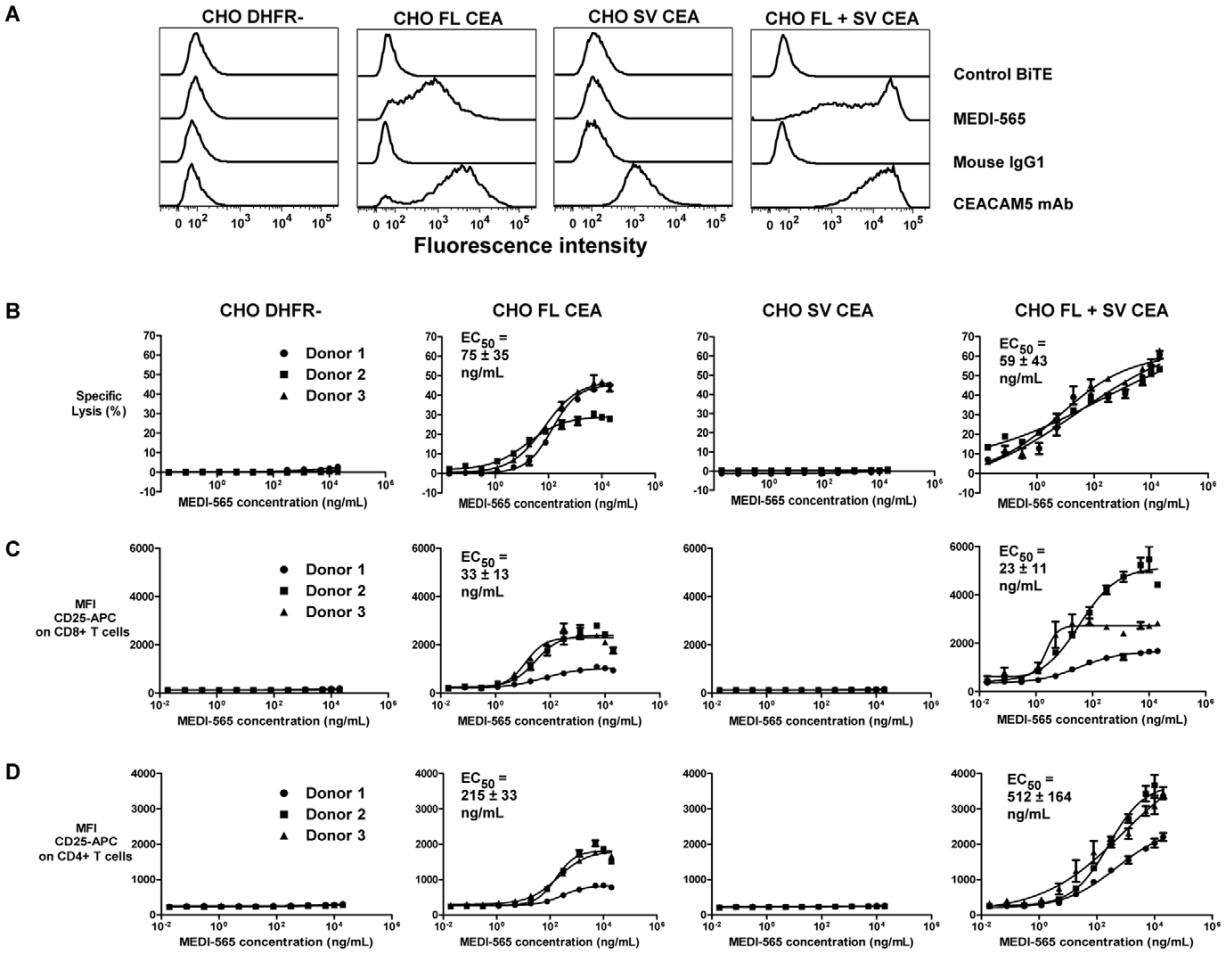
Role of the CEA splice variant in MEDI-565 mediated T cell activation and target cell killing. A, expression of full-length CEA and CEA splice variant proteins in CHO cells as determined by flow cytometry. Mouse IgG1, mouse IgG1 control antibody. B, CHO DHFR-, CHO FL, CHO SV and CHO FL+SV cells were tested for their susceptibility to be killed by CD3+ T cells from 3 individual donors in the presence of MEDI-565 at the indicated concentrations. EC_50_ values listed indicate the mean value among 3 donors±standard error of the mean. p = 0.79 comparing cytotoxicity EC_50_ values between CHO FL CEA and CHO FL+SV CEA cells. C, activation (increased cell surface CD25/IL-2R levels) of CD8+ T cells and D, activation of CD4+ T cells isolated from each of the 3 healthy donors was investigated concurrently with the cytotoxicity assays shown in panel B. p = 0.60 comparing CD8+ T cell activation EC_50_ values between CHO FL CEA and CHO FL+SV CEA; p = 0.15 comparing CD4+ T cell activation EC_50_ values between CHO FL CEA and CHO FL+SV CEA. MFI CD25-APC = mean fluorescence intensity of bound APC labeled, anti-human CD25 mAb. Experiment was repeated once with similar results.

Although the CEA splice variant was infrequently found in pancreatic tumors, it was found at a high frequency in colorectal (98%) and gastroesophageal (50%) cancers, and to a lesser degree in lung (30%) and breast (12%) tumors. Due to its concordant expression with full length CEA in human tumors, we sought to understand the binding of MEDI-565 to the CEA splice variant and the role that this CEA isoform might play in targeting CEA-positive tumors. The amino acids in full-length CEA important for MEDI-565 binding were found to be largely absent in the CEA splice variant, except amino acids I^408^ and N^410^ in segment C (Figure 2). This observation suggested that binding of MEDI-565 to the CEA splice variant was unlikely to occur. To test this hypothesis, CHO cells were infected with lentivirus constructs directing the expression of full-length CEA (CHO FL CEA) or the CEA splice variant (CHO SV CEA), or both concurrently after sequential infection of cells with the CEA splice variant then the full-length CEA (CHO FL+SV CEA); full-length CEA and CEA splice variant protein expression were verified by western blotting (Figure S1). As anticipated, MEDI-565 bound to cells expressing full-length CEA but not to cells expressing the CEA splice variant (Figure 6A). We note that a higher level of MEDI-565 binding was observed for cells expressing both the CEA splice variant and full length CEA together relative to cells expressing only full length CEA. This is likely due to different levels of expression efficiency in each independently infected cell line, although effects of the CEA splice variant protein on full length CEA protein levels or epitope accessibility in cells cannot be ruled out.

Zhou et al [48] showed that the both the N-terminal domain and A3 mediated the interaction between CEA molecues on apposing cell surfaces; both domains are present within the CEA splice variant. Thus, we tested the hypothesis that co-expression of the CEA splice variant and full-length CEA proteins may, through heterophilic interactions, result in the MEDI-565 binding epitope being masked in the full-length form and subsequently prevent or reduce MEDI-565 binding. Co-expression of the CEA splice variant with full-length CEA on the same cells did not significantly affect the apparent binding affinity of MEDI-565 to full-length CEA (CHO FL CEA, apparent K_D_ = 5.0±0.15 nM; CHO FL+SV CEA, apparent K_D_ = 5.6±3.0 nM; p = 0.86). These results were expected since homophilic interactions between full-length CEA proteins also do not appear to prevent the binding of MEDI-565 [17], [18] or other CEA-specific BiTE antibodies [16].

Consistent with the MEDI-565 binding data, CHO SV CEA did not trigger the activation of T cells from healthy donors in the presence of MEDI-565, as measured by the up-regulation of the IL-2Rα chain/CD25 protein on either CD8 or CD4 T cells (Figure 6C and D). This T cell activation marker has been shown previously to correlate temporally with the release of cytokines from T cells activated by MEDI-565[18] and other BiTE antibodies [23], [25]. Likewise MEDI-565 also did not mediate the lysis of target cells expressing the splice variant (Figure 6B). In contrast, MEDI-565 activated T cells and induced killing of CHO FL CEA or CHO FL+SV CEA cells with similar levels of potency (EC_50_ values: CHO FL CEA, EC_50_ = 75±35 ng/mL; CHO FL+SV CEA, EC_50_ = 59±43 ng/mL; p = 0.79). Somewhat higher maximal T cell activation and maximum achievable cytotoxicity levels were observed with CHO FL+SV CEA cells relative to CHO FL CEA cells, and may be explained by the higher full length CEA expression in the former, as described above.

Studies with full length CEA and the splice variant were carried out in CHO cells due to their ease of infection with lentiviral constructs and their subsequent selection of CEA-expressing cells using flow cytometry. To demonstrate the killing specificity of MEDI-565 for CEA positive human tumor cells, we examined numerous human cancer cell lines for CEA expression and for their ability to be killed by MEDI-565 activated T cells. Consistent with the specificity observed for CHO cells, MEDI-565 mediated the killing of human tumor cells that expressed CEA, but not those that did not express cell surface CEA (Figure S2). Additionally, a control BiTE did not induce T cell lysis of CEA positive or negative cell lines.

## Discussion

To provide insights for CEA-targeted diagnostics and therapy and understand the specificity of anti-CEA mAbs, many efforts have been made to characterize the binding epitopes of these antibodies using various approaches [49], [50], [51], [52]. The most extensive investigations involve the classification of 52 anti-CEA mAbs into five distinct epitopes, Gold epitopes 1–5, by competitive binding analysis [50]. Further studies have correlated the Gold epitopes to the domains of CEA using different CEA fragment constructs [51], [53], [54], however there is no precise localization of the epitopes to the amino acid sequence level. An attempt to identify the amino acid sequences corresponding to Gold epitopes using synthetic overlapping fifteen-mer peptides has failed [55], suggesting that the Gold epitopes are conformational. In addition, immunohistochemistry and immuno flow cytometry were used to demonstrate a good correlation between the Gold epitope groups and their binding specificity: 1) mAbs that bind to Gold epitopes 2 and 3 were generally specific to CEA, reacting only with colon carcinoma, normal colon mucosa and normal gastric foveola; 2) mAbs in epitopes 4 and 5 were highly cross-reactive with different normal tissues possibly due to binding to CEA-related antigens; and 3) both specific and cross-reactive mAbs were found in epitope 1 [56]. We have mapped the epitope of the CEA-specific arm of MEDI-565 to the A2 domain comprised of two stretches of amino acids, 326–349 and 388–410. Interestingly, the MEDI-565 epitope on CEA belongs to the Gold epitope 2, which has been localized in the A2-B2 domains [53] and identified as a CEA-specific epitope group [56].

CEA comprises highly repetitive immunoglobulin domains of A1-B1, A2-B2, and A3-B3. Some anti-CEA mAbs bind repetitive epitopes of CEA [51] and some are highly cross-reactive, lacking specificity to CEA [56]. The anti-CEA arm of MEDI-565 is a humanized version of the murine antibody A5B7, which has been shown to specifically bind to CEA [40], [56]. Characterizing the epitope of MEDI-565 has led us to propose that the critical residues F^326^, T^328^, and N^333^ mediate the fine specificity to CEA of MEDI-565 due to their uniqueness in the A2 domain of CEA when compared with all other immunoglobulin domains of CEA and other related molecules of the CEACAM family, including CEACAM1, CEACAM3, CEACAM4, CEACAM6, CEACAM7, and CEACAM8.

After identifying the binding epitope of MEDI-565, we evaluated the impact of polymorphisms and isoforms in the A2 domain of CEA on the activity of MEDI-565. Two non-synonymous SNPs were identified in the binding epitope of MEDI-565 but occurred at a very low frequency in the general population and were considered to have little or no impact on MEDI-565 binding to CEA. In addition, a single splice variant was identified lacking a portion of the A2 domain critical for MEDI-565 binding. Efforts to understand the association of splice variant expression to disease found that its expression occurred in a substantial percentage of primary human colorectal and gastroesophageal tumors, and to a lesser extent in lung and breast tumors in a pattern that was concordant with full length CEA expression. However, since MEDI-565 did not bind the CEA splice variant due to loss of the antibody-binding epitope, it was clear that the splice variant itself is not a target for MEDI-565 in primary human tumors that also express full length CEA. Because CEA could participate in homotypic interactions on adjacent cells, it remained possible that expression of the CEA splice variant may interfere with the binding of MEDI-565 and the subsequent tumor cell lysis through its interaction with full length CEA. By co-expressing the CEA splice variant with full length CEA, we formally demonstrated that this was not the case, as MEDI-565 binding to full length CEA and potency of CEA-directed lysis were not significantly affected by simultaneous expression of the splice variant and full length CEA. Therefore, the expression of the CEA splice variant by primary human tumor cells is not anticipated to interfere with MEDI-565 binding to full-length CEA, nor should it inhibit MEDI-565-mediated T-cell killing of tumor cells expressing full-length CEA. However, discrimination of full-length CEA from the CEA splice variant may be important while monitoring the status of CEA positive tumors via changes in serum CEA levels in clinical study patients that receive MEDI-565.

## Supporting Information

Figure S1 Western blot of CEA protein expressed by CHO cell lines. The full-length CEA protein is indicated by a filled arrowhead, and the CEA splice variant by an open arrowhead; both proteins were detected using a CEACAM5-specific mAb. Molecular weights (kilodaltons; KDa) of the protein standard are indicated to the left of the image. Lanes of CHO FL and CHO FL+SV cell lysates contain a band at ∼100 kDa that is presumed to be a non-glycosylated form of CEA. Equal amounts of protein loaded into each lane of the gel were controlled by detecting GAPDH. CHO DHFR-, parental CHO cells; CHO FL CEA, full-length CEA-expressing CHO; CHO SV CEA, CEA splice variant expressing CHO; CHO FL+SV CEA, CHO cells co-expressing full-length and splice variant CEA.
(TIF)

Figure S2 MEDI-565 mediated binding to and T cell lysis of CEA positive, but not CEA negative, human cancer cell lines. A, flow cytometry analysis of MEDI-565 binding to CEA positive (H727 and HPAF-II) and CEA negative (A2780 and Colo205) human cancer cell lines; MFI, mean fluorescence intensity of AlexaFluor® 488 anti-penta-His secondary antibody bound to MEDI-565. B, Lack of CEA-negative tumor cell killing by T cells engaged by MEDI-565 or control BiTE. Percent control represents degree of specific cell killing, as measured by release of cellular caspase 3, above that of untreated T cells plus target cells (set at 100%). C, Killing of CEA positive tumor cells by MEDI-565 but not by the control BiTE.
(TIF)

Table S1 Ct values for full-length (FL) CEA and CEA splice variant (SV) cDNA expression in A, pancreas; B, colon; C, breast; D, lung; E, gastrointestinal tissues.
(PPT)
