# Supplementary figures and images for: The CEA/CD3-Bispecific Antibody MEDI-565 (MT111) Binds a Nonlinear Epitope in the Full-Length but Not a Short Splice Variant of CEA
Source: PLoS One. 2012 May 4;7(5):e36412. doi: 10.1371/journal.pone.0036412 (PMC3344869; doi:10.1371/journal.pone.0036412)

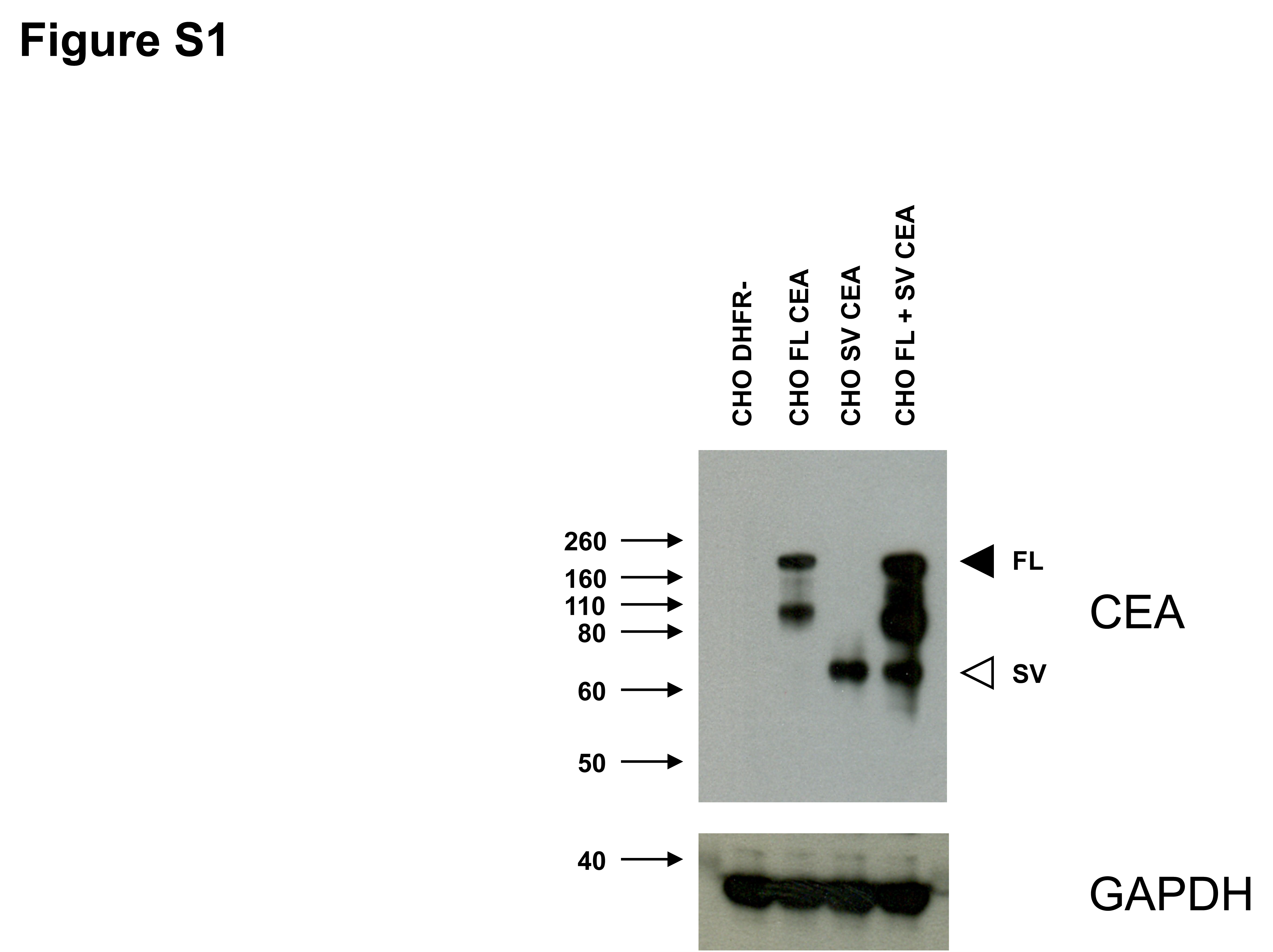

Supplement: Figure S1 — Western blot of CEA protein expressed by CHO cell lines. The full-length CEA protein is indicated by a filled arrowhead, and the CEA splice variant by an open arrowhead; both proteins were detected using a CEACAM5-specific mAb. Molecular weights (kilodaltons; KDa) of the protein standard are indicated to the left of the image. Lanes of CHO FL and CHO FL+SV cell lysates contain a band at ∼100 kDa that is presumed to be a non-glycosylated form of CEA. Equal amounts of protein loaded into each lane of the gel were controlled by detecting GAPDH. CHO DHFR-, parental CHO cells; CHO FL CEA, full-length CEA-expressing CHO; CHO SV CEA, CEA splice variant expressing CHO; CHO FL+SV CEA, CHO cells co-expressing full-length and splice variant CEA. (TIF) [file pone.0036412.s001.tif]

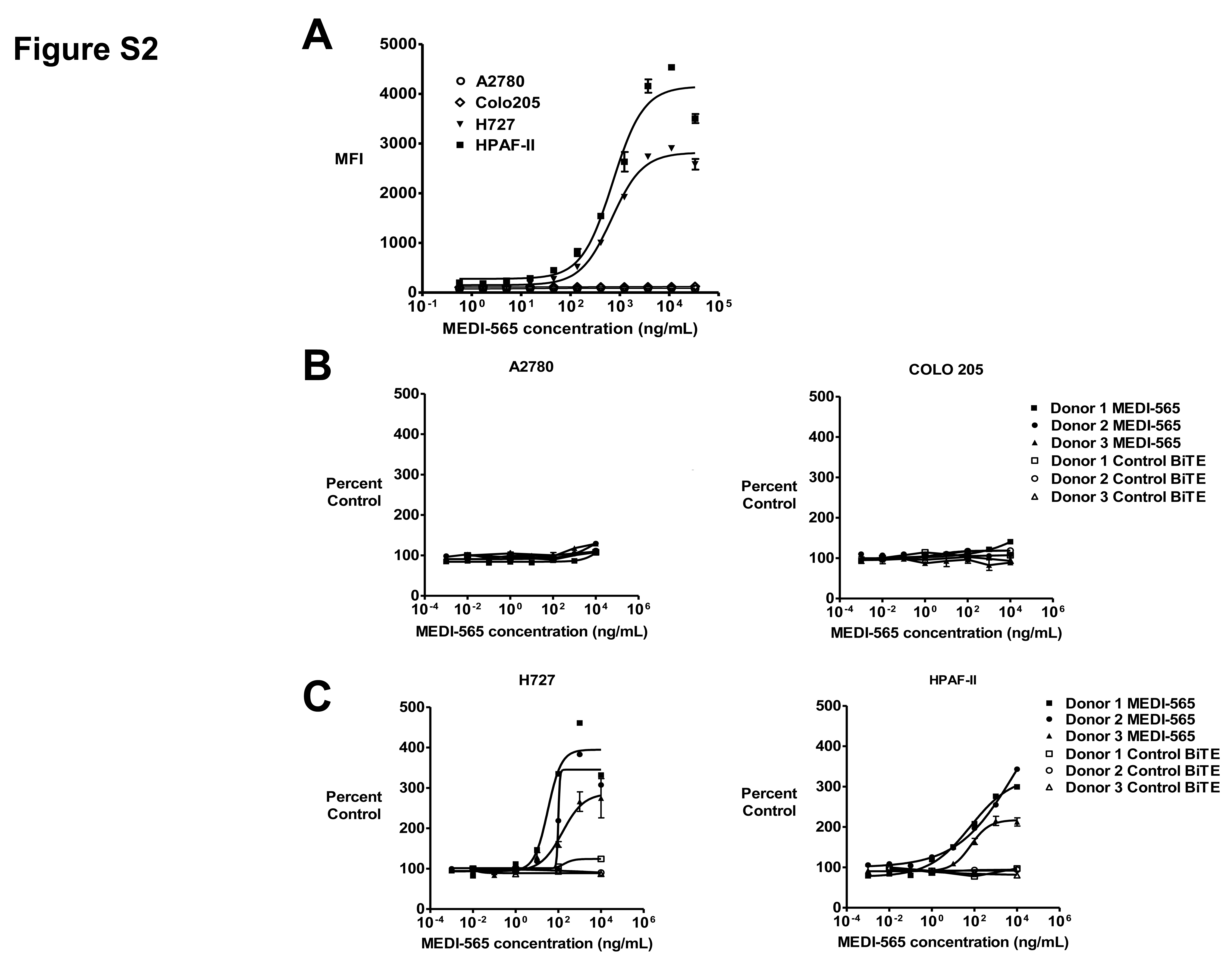

Supplement: Figure S2 — MEDI-565 mediated binding to and T cell lysis of CEA positive, but not CEA negative, human cancer cell lines. A, flow cytometry analysis of MEDI-565 binding to CEA positive (H727 and HPAF-II) and CEA negative (A2780 and Colo205) human cancer cell lines; MFI, mean fluorescence intensity of AlexaFluor® 488 anti-penta-His secondary antibody bound to MEDI-565. B, Lack of CEA-negative tumor cell killing by T cells engaged by MEDI-565 or control BiTE. Percent control represents degree of specific cell killing, as measured by release of cellular caspase 3, above that of untreated T cells plus target cells (set at 100%). C, Killing of CEA positive tumor cells by MEDI-565 but not by the control BiTE. (TIF) [file pone.0036412.s002.tif]
