# Supplementary material for: The CEA/CD3-Bispecific Antibody MEDI-565 (MT111) Binds a Nonlinear Epitope in the Full-Length but Not a Short Splice Variant of CEA
Source: PLoS One. 2012 May 4;7(5):e36412. doi: 10.1371/journal.pone.0036412 (PMC3344869; doi:10.1371/journal.pone.0036412)
Supplement: Table S1 — Ct values for full-length (FL) CEA and CEA splice variant (SV) cDNA expression in A, pancreas; B, colon; C, breast; D, lung; E, gastrointestinal tissues. (PPT) [file pone.0036412.s003.ppt]

## Slide 1
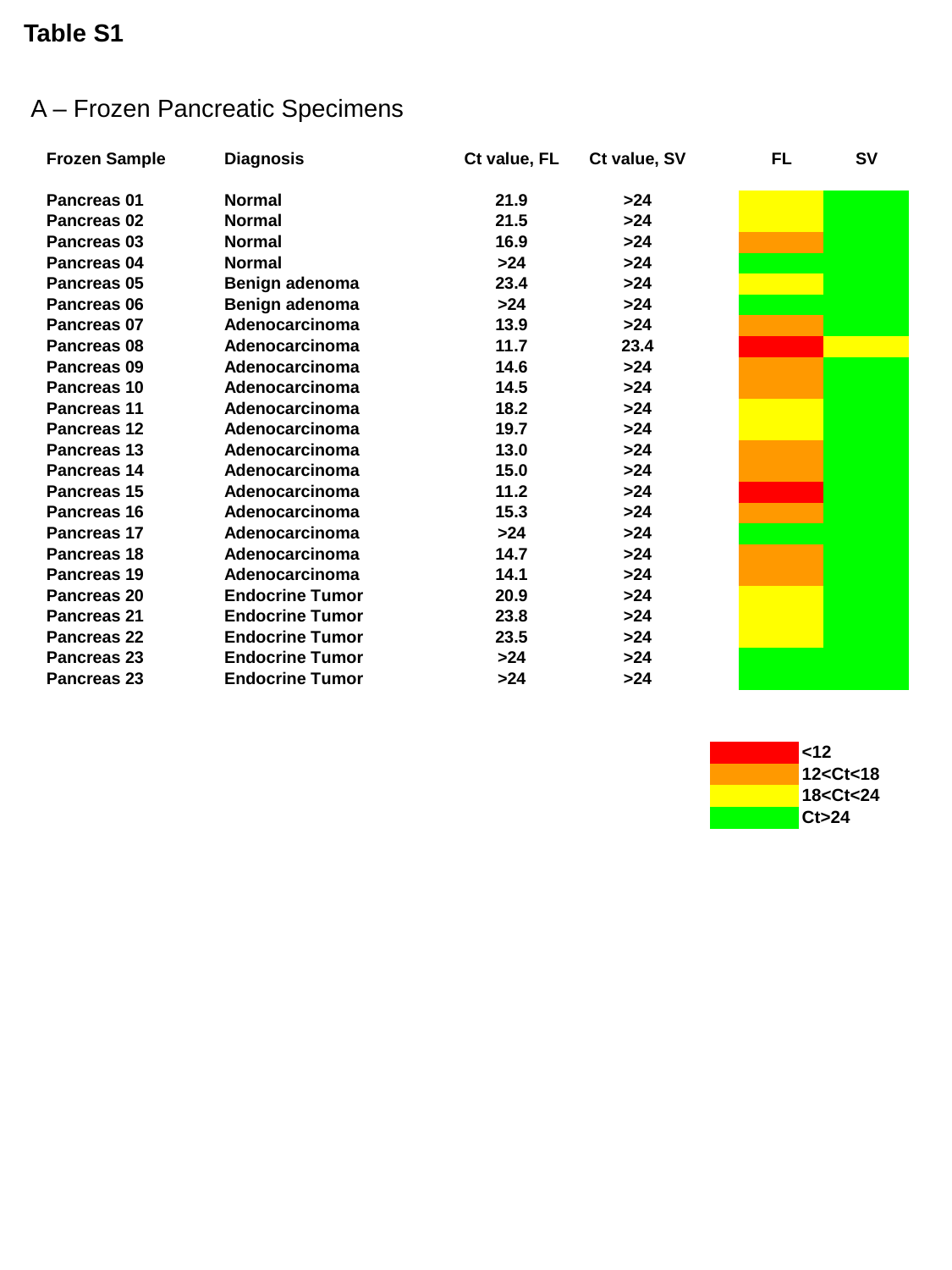

Table S1
A – Frozen Pancreatic Specimens

## Slide 2
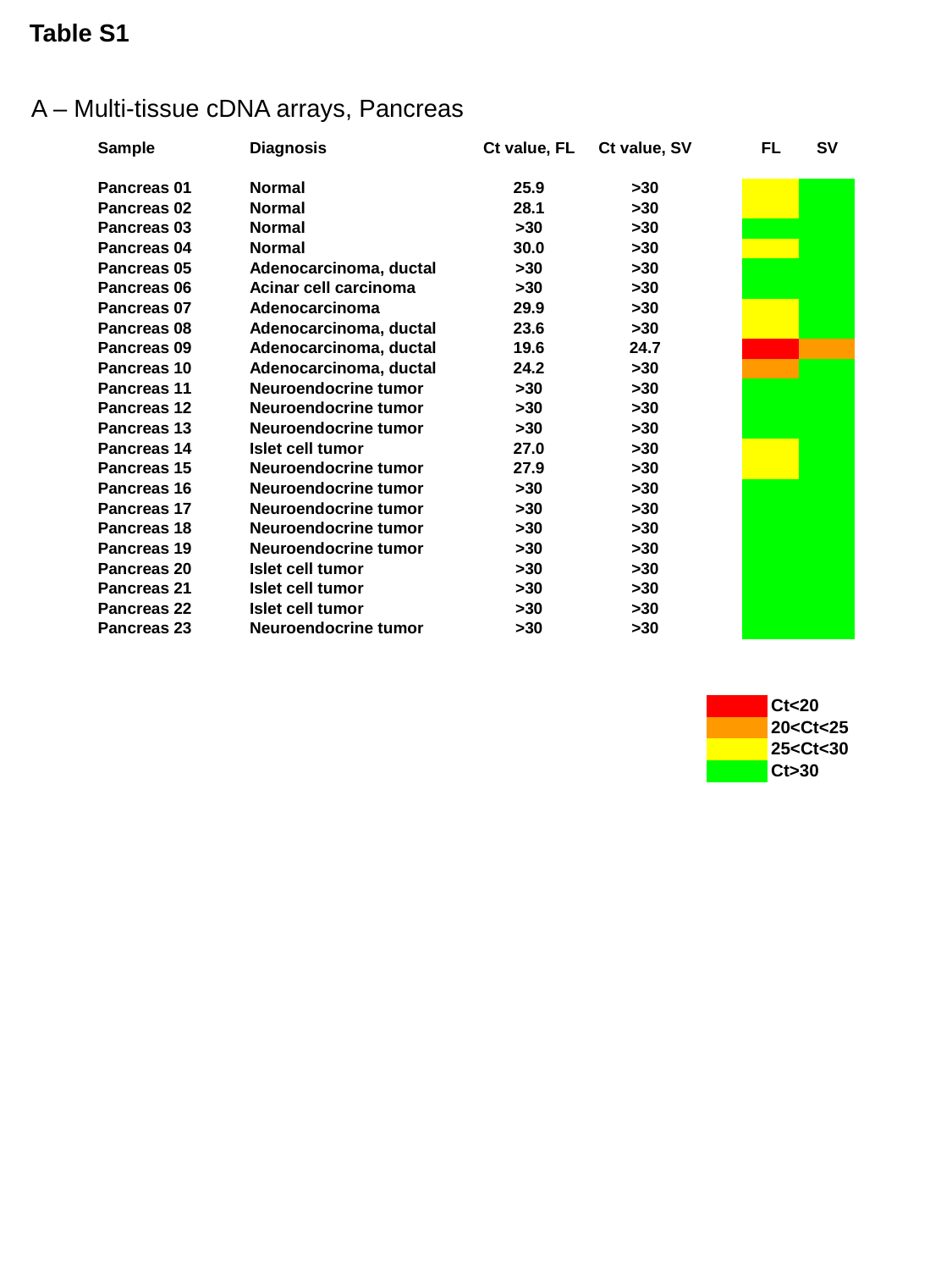

Table S1
A – Multi-tissue cDNA arrays, Pancreas

## Slide 3
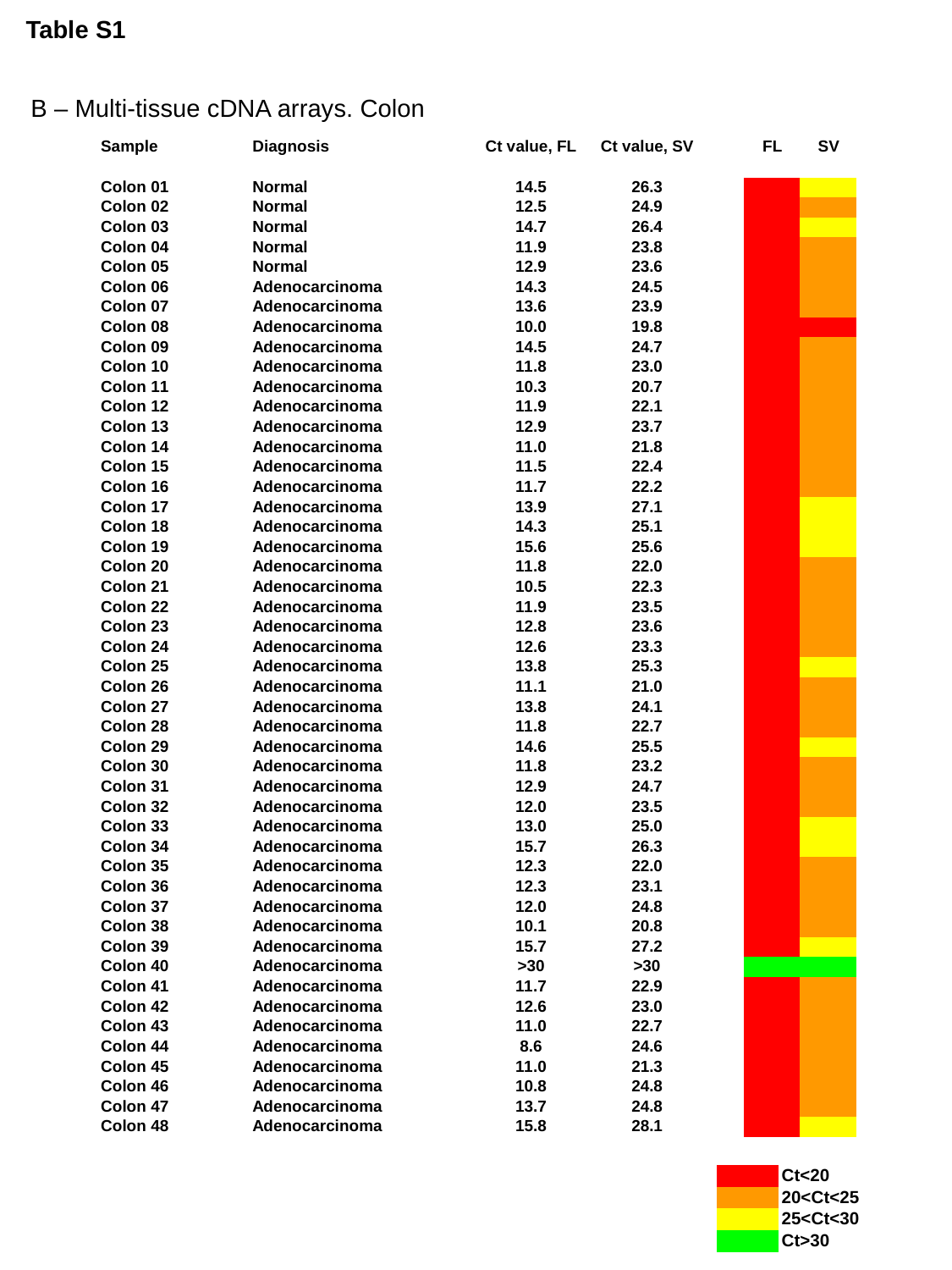

Table S1
B – Multi-tissue cDNA arrays. Colon

## Slide 4
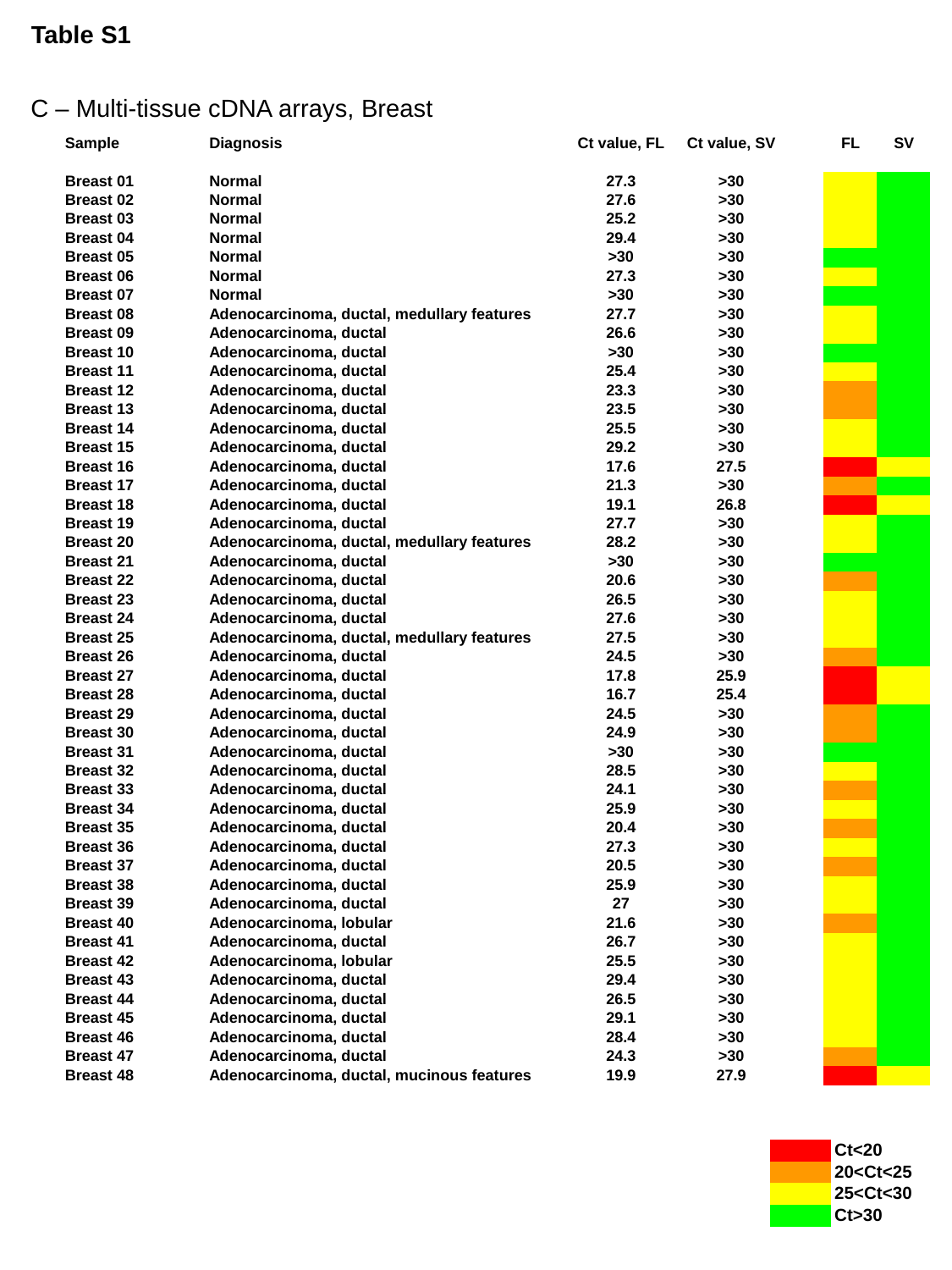

Table S1
C – Multi-tissue cDNA arrays, Breast

## Slide 5
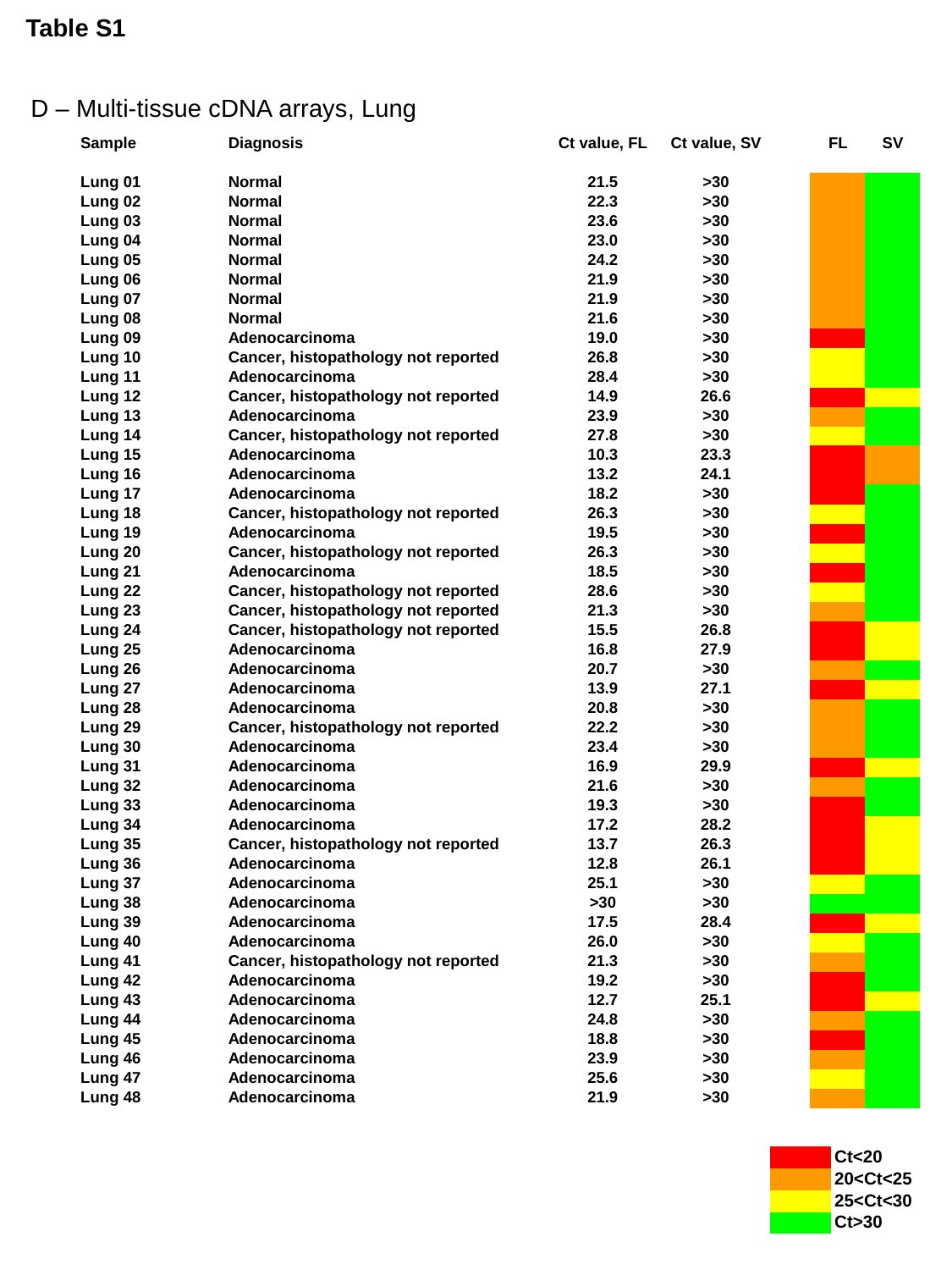

Table S1
D – Multi-tissue cDNA arrays, Lung

## Slide 6
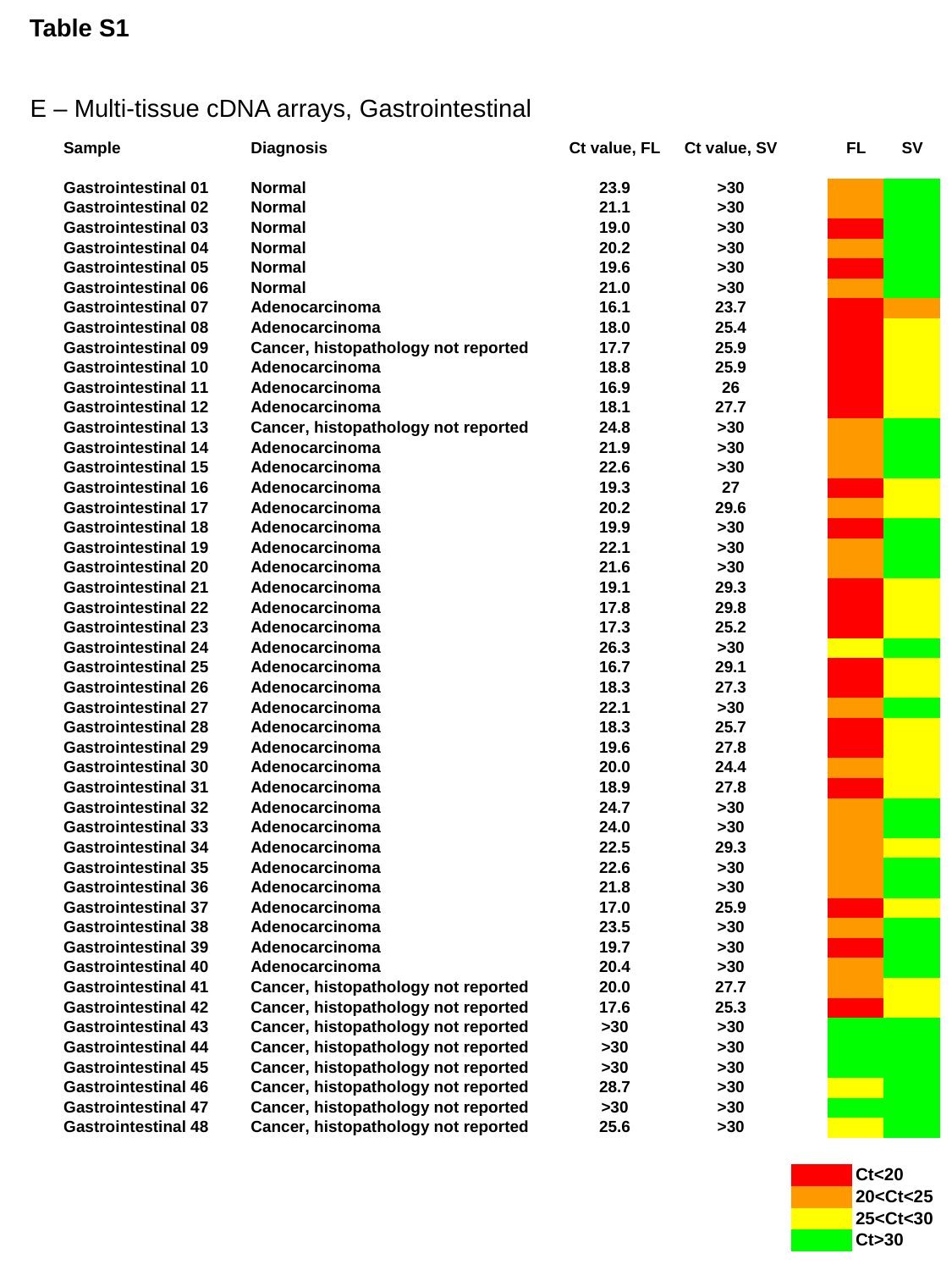

Table S1
E – Multi-tissue cDNA arrays, Gastrointestinal
